# Experimentally investigating the origin of DNA/RNA on early Earth

**DOI:** 10.1038/s41467-018-07212-y

**Published:** 2018-12-12

**Authors:** Ramanarayanan Krishnamurthy

**Affiliations:** The Scripps Research Institute, 10550 North Torrey Pines Road, La Jolla, CA 92037 USA

## Abstract

There are varied views about how the molecules of life may have appeared on early Earth. Nowhere is this divergence more acute than in the origins of DNA/RNA and has become a matter of constant deliberations.

## Introduction

**Fig. 1:**
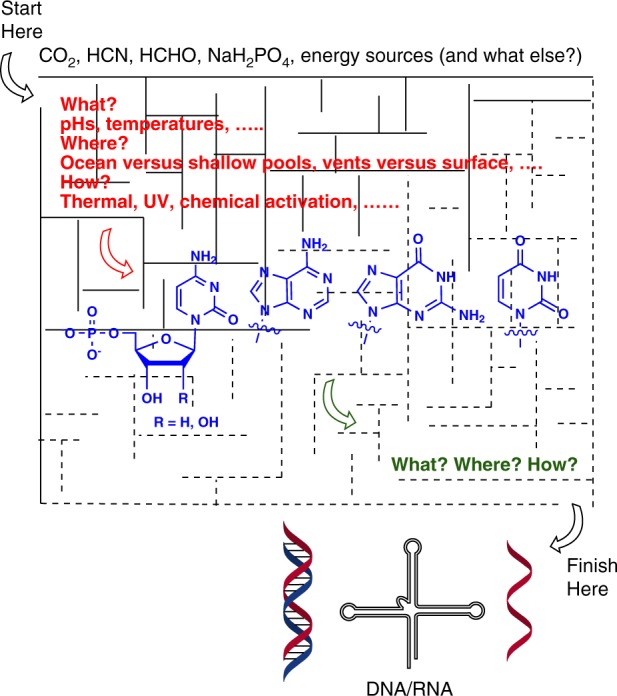
Exploring the maze of conditions leading to formation of nucleotides and eventually to DNA/RNA. The end-goal of getting DNA and RNA from a given set of plausible starting materials should seem straightforward but is complicated by the labyrinth of prebiotically plausible conditions and environments on early Earth

Understanding the chemical origins of DNA and RNA in the context of origins of life continues to be an enigma, which has spawned a plethora of investigations of plausible pathways (Fig. 1)^1^. Understandably, each of these investigations is rooted in deep convictions—partly based on scientific facts and the remaining filled with extrapolated scientific imaginations about the early Earth scenarios and the availability of source materials that could/would lead to the building blocks of, and eventually to, DNA and RNA. And it is this extrapolated scientific imaginative part that creates difficulties in providing definitive answers to the question, “What were the conditions on Early Earth when nucleotides were formed and what are the most plausible nucleoside candidates? In other words: Which experimental conditions should be chosen when designing an experiment to investigate the origin of DNA/RNA?” Before we attempt to comment, it would be prudent to remind ourselves of a refrain from the Keno Upanishad: “It is known to him to whom it is unknown; to whom it is known he knows not. To those who know, it is unknown; to those who do not know, it is known.”^2^. In other words, we must ask ourselves whether we have and know all the scientific facts necessary (or lack thereof) to answer the above question.

There is a good reason as to why the scientific imaginative part exists—it stems from a lack of consensus^3^, a lack of a firm understanding and good experimental evidence of “what were the conditions on early Earth”, leading to uncertainty about “which experimental conditions should be chosen” (Fig. 1). While there are some common ground on what would be needed for organic synthesis of DNA/RNA (for example, the components of ribose and nucleobases to come from formaldehyde, cyanide and their derivatives), there is still a fair amount of uncertainty associated with what is defined and *universally accepted* as prebiotically plausible conditions^4,5^. There seems to be no unanimity among the geologists, geochemists and geobiologists as to what the early earth conditions were that would foster environs for self-sustaining prebiotic chemistries, even though there have been many attempts at reconstruction of the early Earth conditions^6^. Reasons for disagreement range from the difficulties faced in interpreting early rock/fossil records based on how well they are preserved, to doubts in ruling out contamination from extant life, to errors introduced by analytical techniques (on miniscule amount of samples)^7^. The resulting ambiguity has fueled widely different ideas from different research groups on plausible prebiotic conditions to investigate the origin of DNA/RNA—and this ambiguity applies equally to “what are the most plausible nucleoside candidates”, that could be pre-RNA/pre-DNA. Lacking specific experimental evidence, the prebiotic chemists have taken the lead, guided by clues mainly based on chemical reactivity (either from organic chemistry or biochemical pathways) to synthesize the nucleosides and nucleotides of DNA/RNA. Chief among them are the still sought-after ‘direct-condensation-approach’ of ribose (or its derivatives) with the fully formed nucleobases (e.g. adenine) or the currently (more) favored ‘building-the-sugar-unit-along-with-the-base’ approaches^1,8,9^. While neither of these approaches has found universal acceptance within the origins of life community at large, the search for other plausible nucleoside candidates have surged to the fore^10^, however, with equal resistance to universal acceptance—with an added burden of having to explain the exact pathways by which they would give rise to DNA/RNA^11^.

Thus, barring the discovery of ‘chemical fossils’ from preserved prebiotic pathways on early Earth with which we can check and confirm—all of our approaches to understanding the chemical origin of DNA/RNA, could be, at best, classified as “inventions”, which in itself is a welcome realization, a “reality-check”. As succinctly summed up by Eschenmoser “*Origin of Life cannot be discovered; it has to be* *reinvented*”^12^. And, this need for ‘reinvention’ allows us the necessary leeway for reasonably hypothesizing and investigating a library of prebiotically plausible source materials and conditions that need to be (and should be) examined and screened in order to assess what is feasible (or not). This ‘reinvention’ can provide quite refreshing solutions to the previously notorious nucleosidation problem of directly reacting canonical purine and pyrimidine bases with ribose, where all attempts to date have proven unproductive^1^. The solutions range from the pioneering work of Orgel’s ‘building-the-base-on-the-sugar’ approach^1^ that has been taken even further to elementary building blocks by Powner and Sutherland^13^, to the Eschenmoser approach^14^ of building the purines via the 5-formamidopyrimidine nucleosides that has been brought to fruition by Carell and co-workers^9^, to the direct nucleosidation with ribose-1,2-cyclophosphate by Benner and colleagues^15^—to mention a few. These wide-ranging solutions are under very different conditions that do not, at first or even second glance, seem to be compatible with one another; however, that should not be (or be made) the issue. What is important to note is that a range of plausible prebiotic pathways starting from various building blocks under various reaction conditions do provide some idea of what is abiotically feasible. Of course, each of these approaches does have its detractions in terms of what is “prebiotically plausible”, and this takes us back to the very beginning of how little we know—or to be precise—how much we do not know about early Earth conditions to make definitive judgments.

Going back to the two points raised at the beginning in the context of early conditions on earth, (a) the most plausible nucleoside candidates and (b) the experimental conditions to be chosen, it becomes apparent that there can be no single solution, since there is ambiguity in the geochemical and geophysical constraints or chemical evidences that would allow for a firm ‘yes—these are the Early Earth conditions’. While, there are some constraints where there is almost complete agreement about what is plausible, there are large domains where there is lack of consensus^3,4,6^. For example, there is a large body of evidence about the existence of anoxic atmosphere on early Earth^4,6^ (that is the lack of oxygen), which has consequences for chemistry—such as the use of Fe^2+^ (and not Fe^3+^) in prebiotic chemical reactions. However, there are many other parameters where it is less clear (pH, temperature, ocean versus shallow pools, vents versus surface, the necessity for ultraviolet radiation versus protection from it etc. etc.), and how these conditions could lead to self-sustained availability of activated chemical precursors at concentrations that would transform themselves to DNA/RNA^3,4,6^. There are more unknowns than knowns, which makes it all the more challenging to state, “yes, these are the early earth conditions, these are plausible nucleoside candidates, and these are the experimental conditions to be chosen” (Fig. 1). This unresolved state permits each experimental approach to pick early Earth conditions tailored to its demonstrated synthetic pathways and stake the claim of relevance. One certainly can propose them, but only with the appreciation that they are broad approximations that can, and will, be challenged before “the ink is dry” on the publication.

Notwithstanding this, the various approaches have over the years led to preferences among the practitioners, because either they are genuinely convinced that it is the only plausible solution or perhaps due to the psychological fact that they have discovered reactions that are, understandably, close to their heart and beliefs. A case-in-point is our own work on the diamidophosphate (DAP) mediated phosphorylation chemistries^16^, where we are currently ‘convinced’ that nucleotide formation and oligomerization towards DNA/RNA (or alternative systems) could be mediated by nitrogenated phosphorus derivatives, and not necessarily by phosphates, as has been the case (mostly) thus far^17^. This is, understandably, a ‘bias’ that is currently driving many of the investigations in our laboratory—as (reasonably) is the case with many of the groups trying to understand the chemical origins of DNA/RNA and/or the alternative nucleosides. Therefore, it is not surprising that this endeavor (like any other in the Origins of Life) would generate different viewpoints and can become a point of contention and controversy as each investigator considers their conditions and scientific imaginations to be the most prebiotically plausible. While this situation could be viewed as suboptimal, it could also be—in an optimistic view—a source of strength, since the diverse approaches demonstrate the ‘robustness’ with which various potential prebiotic pathways and claims can be not only demonstrated abiotically, but also examined and judged for being useful (or useless) in the quest for understanding the chemistry that led to the origins of DNA/RNA (Fig. 1)^5^. It is part of a healthy scientific debate, questioning the various approaches based on the perceived weaknesses and strengths with respect to the differing scenarios.

For many of us who, perhaps, were uncomfortable reading the mystically sounding statement from the Keno Upanishad in the beginning of the commentary when trying to answer the question posed, we may be reassured by the following statement—as a scientific equivalent—from a pioneer and an impeccable and quintessential practitioner of prebiotic chemistry, Leslie Orgel, who said “Anybody who thinks they know the solution to this problem (of the origins of life) is deluded. But, anybody who thinks this is an insoluble problem is also deluded”^18^. Therefore, with the ongoing and forthcoming advances in this field, we can continue our ‘delusional’ state in searching for ‘definitive answers’, and with each publication continue to accumulate the knowledge to appreciate how much we still don’t (won’t?) know!
